# 

**Published:** 1889-03-23

**Authors:** 


**PERMANENTLY ENLARGED TO 32 PAGES.**

PRICE ONE PENNY.

No. 130, Vol. V.—March 23rd, 1889.

[Registered at the General Post Office as a Newspaper.]

Per Annum, 6s. 6d. post free.
Monthly Parts, 6d.; post free, 7½d.
Ditto per Ann. 7s. 6d., post free.

### THE HOSPITAL

A WEEKLY INSTITUTIONAL JOURNAL OF

